# Supplementary material for: An optical neural chip for implementing complex-valued neural network
Source: Nat Commun. 2021 Jan 19;12:457. doi: 10.1038/s41467-020-20719-7 (PMC7815828; doi:10.1038/s41467-020-20719-7)
Supplement: Supplementary file 1 — Supplementary Information [file 41467_2020_20719_MOESM1_ESM.pdf]

SUPPLEMENTARY INFORMATION FOR

An Optical Neural Chip for Implementing Complex-Valued Neural Network

H. Zhang[1], M. Gu[2,3], X. D. Jiang[1*], J. Thompson[3], H. Cai[4], S. Paesani[5], R. Santagati[5], A. Laing[5], Y. Zhang[1,6], M. H. Yung[7,8], Y. Z. Shi[1], F. K. Muhammad[1], G. Q. Lo[9], X. S. Luo[9], B. Dong[9], D. L. Kwong[4], L. C. Kwek[1,3*], and A. Q. Liu[1*]

[1] *Quantum Science and Engineering Centre (QSec), Nanyang Technological University, 50 Nanyang Ave, 639798, Singapore*

[2] *Complexity Institute and School of Physical and Mathematical Sciences, Nanyang Technological University, 50 Nanyang Ave, 639798, Singapore*

[3] *Centre for Quantum Technologies, National University of Singapore, Block S15, 3 Science Drive 2, 117543, Singapore*

[4] *Institute of Microelectronics, A*STAR (Agency for Science, Technology and Research), 138634, Singapore*

[5] *Centre for Quantum Photonics, H. H. Wills Physics Laboratory and Department of Electrical and Electronic Engineering, University of Bristol, Merchant Venturers Building, Woodland Road, Bristol BS8 1UB, UK*

[6] *School of Mechanical & Aerospace Engineering, Nanyang Technological University, 50 Nanyang Ave, 639798, Singapore*

[7] *Institute for Quantum Science and Engineering, Department of Physics, Southern University of Science and Technology, Shenzhen 518055, China*

[8] *Shenzhen Key Laboratory of Quantum Science and Engineering, Southern University of Science and Technology, Shenzhen 518055, China*

[9] *Advanced Micro Foundry, 11 Science Park Road, 117685 Singapore*

**Corresponding Authors: exdjiang@ntu.edu.sg, cqtklc@nus.edu.sg, eaqliu@ntu.edu.sg*

Supplementary Note 1 – Related Works

We discuss the contributions of some previous works related to complex-valued neural networks and clarify the salient differences between their schemes and our implementation.

1. A diffractive deep neural network[1] which discusses complex-valued network has been reported. However, it requires spatial light modulators or 3D-printed diffractive elements which are not conducive to integration. And the 3D-printed classifiers are fixed once fabricated whereas our optical neural chip is based on photonic chips with reconfigurable classifiers.
2. Another optical neural network accelerator based on photoelectric multiplication was reported[2]. Unlike the previously mentioned approaches, this scheme encoded both weights and inputs optically, and the matrix multiplication was realized by combining the input and weight signals for balanced homodyne detection. Although involving coherent detection, the network architecture applicable to the accelerator was still real-valued, because the optical signals had already been converted to photocurrents by the detectors before reaching the accumulator ($\sum_{j=1}^{n}|A_j||x_j|$ vs. $\sum_{j=1}^{n}|A_j||x_j|e^{i(\phi_A+\phi_x)}$, where $A_j=|A_j|e^{i\phi_A}$ and$x_j=|x_j|e^{i\phi_x}$). Motivated by the parallelization capability of this scheme, an architecture using wavelength division multiplexing for vector-matrix multiplication was proposed[3]. This architecture exploits optical parallelization and shows promise for low-power and low-latency optical neural networks. However, their goal was distinct from ours in seeking to realize parallel computing while we are actually designing a complex neural network.
3. Complex-valued reservoirs were reported in reservoir computing for enriching the system dynamics and improve the performance[4]. Corresponding training methods of

the reservoirs' read out weights were investigated[5]. However, the reservoir computing are not general neural networks, as the internal dynamics of reservoirs are uncontrollable, while in a neural network we expect to control the weight matrices. Moreover, a neural network is known as universal approximator of any mathematic function[6], while reservoir computing has no such guarantee.

Supplementary Note 2 – Phase Shifter Characteristics

The MZIs are modulated by thermo-optic effect. The thermal is provided by the integrated heater. The designed waveguide cross section is shown in Figure 1. The waveguide is 450×220 nm^2. The TiN heater has length of 100 μm, width of 3 μm and thickness of 120 nm. The distance between TiN heater and the top of waveguide is 2 μm. The thermo-optic coefficient of silicon is $\frac{dn}{dT} = 1.86 \times 10^{-4}$ RIU/°C . From our experimental results, the chip-integrated heater is a slightly non-resistive load as observed from the I-V characteristics shown in Figures 2a and 2b. Therefore, the relationship between the actual electric power and the current applied to the heater is fitted polynomial.

The power consumption of the heater is summarized as: Theoretically, the voltage and current required to drive the heater for a 2π phase shift is 5 V and 15 mA. From our experimental results, the equivalent resistance of each heater is about 342.8 Ω, and thus the electrical resistivity is 1.25×10^{-6} Ω·m. The average electrical power required for a 2π phase shift is 70 mW (14.1 mA). In our new batch of designs, by increasing the length of TiN heaters and digging deep trenches to the Si layer, the required electrical power required for 2π phase shift is reduced to 6.1 mW (3.2 mA, 600 Ω). Notably in experiments, not all phase shifters are working at a full load. Once the chip is trained, the electrical power

applied on phase shifters remain as standing costs. It's proposed that the standing costs can be further reduced[7, 8] by adopting zero-static-power phase change materials[9, 10] or ultra-low power MEMS phase shifters[11, 12]. Besides, in each of the case we reported, the complex-valued algorithm requires fewer neurons and layers, thus less computational resources and power consumptions, compared to the real-valued algorithms.

The modulation frequency of thermo-optic phase shifters reaches up to 10 kHz. The weight matrices are fixed once the training of the network is completed, indicating that we do not need to modulate the phase shifters anymore. The demonstrated chip is faster compared to electronic computer, as discussed from two working conditions. One is the static condition in which the machine learning model is trained and implemented onto the chip. The chip becomes passive and application-specified with all components remain static. The computation speed will not be limited by the modulation rate. And the most time-consuming part of the multiply-accumulate operations is accomplished optically. The other is the dynamic condition in which the modulators are working. Carrier depletion modulators with the typical rate of tens of GHz[13-15] could be a promising alternative. For a complex-valued neural network with dimension of $N \times N \times L$, $4N^2$ multiply-accumulate operations are required for each layer under matrix representations. Therefore, when integrated with 10 GHz carrier-depletion modulators, the ONC can perform $4N^2 \times L \times 10^{10}$ MAC/s. For a single layer ONC with $N = 100$, 10^{14} MAC/s is achieved, which can be benchmarked against to the conventional CPU (10^{11} FLOPS) and advanced GPU (10^{12} FLOPS)[16]. FLOPS stands for floating point operations per second.

One significant consideration of using high-speed modulators is the requirement of advanced electrical interface, such as the RF I/O and high-speed ADCs, TIAs, but is

achievable. A 60-GHz complete transmitter and receiver on System-in-Package (SiP) was demonstrated a few years ago[17]. Although a 100-GHz modulator is still challenging at the moment, realizing a 10-GHz RF Transmitter and receiver along with modulators on SiP no longer presents a technical challenge. Now commercial products for complete single-chip solution for 10 GHz (VYYR2401), 60 GHz (IWR6843AoP, IWR6843) and 77 GHz (IWR1843, IWR1642) are available in the market. High-speed modulators have already successfully served in many speed-demanding applications, such as central processing unit (CPU) – memory interconnects[18] and data centers[19, 20].

Supplementary Note 3 – Thermal and Electrical Crosstalk

Isolation trenches are adopted to improve the heating efficiency and reduce the crosstalk between adjacent heaters. The main contribution of isolation trench is that it improves the heating efficiency. We also conduct the investigation about how trenches help reduce thermal crosstalk. The results are shown in Figure 3. The calibration is done as follows. First, we name the heater in calibration as c-heater and its neighbor heater as n-heater. We assume that the heat from n-heater will spread to the c-heater, resulting in its calibration curve left-shifted. When no electrical power is supplied onto the c-heater, its phase shift will be induced merely by the crosstalk. By increasing the electrical power applied on n-heater, we could get a calibration curve of the effect of crosstalk on c-heater. The isolation trench is proved to be effective in reducing thermal crosstalk. However, the heat accumulation during operation of all phase shifters could lead to potential problems that could not be solved by using isolation trenches alone. To address this problem, we adopt a cooling system for the heat dissipation, as described also in Methods.

Electrical crosstalk usually take place when multiple phase shifters share a common ground. We investigate a simplified model where *n* phase shifters with effective resistance of R_1 are connected to the same ground, and the inevitable wire resistance is denoted as R_2. Consequently, the effective resistance R_{eff} between the supplied voltage V_{cc} and the ground is $R_{eff} = \frac{R_1}{n} + R_2$. The actual electrical power supplied on each phase shifter is $P_{1,act} = \frac{R_1 V_{cc}^2}{(R_1+nR_2)^2}$, while we expect $P_{1,exp} = \frac{V_{cc}^2}{R_1}$. Therefore, the parasitic resistor in the circuit $R_p = 2nR_2 + \frac{n^2 R_2^2}{R_1}$. Thus, if one uses voltage driver, he should make sure that $R_2 \ll R_1$ or accurately calibrate R_2. In contrast, current drivers avoid such annoying problems by providing electrical power $P_{1,act} = P_{1,exp} = I_{cc}^2 R_1$. Note that the current going through the wire resistor R_2 is nI_{cc}, which should be within the stand of current of the connecting wires. We adopt current drivers rather than voltage drivers. To ensure that the current does not exceed the capacity of the circuit, our chip is designed to have every four phase shifters sharing a common ground.

The electrical interface of the optical neural chip is shown in Figure 4. The heaters are supplied by a multi-channel current source. The maximum current of each channel is 24 mA with a resolution of 16 bit, meaning that the minimum step is 370 nA. With all channels in operation, the maximum current from each channel is 15 mA. In data acquisition, the TIA for amplifying the current signal from the PD offers a tunable gain from 1.5×10^4 to 1.5×10^6 V/A. The DAC in use has a detection range of ±10V and a sampling rate of 100 Ks/s/ch simultaneously at 16-bit resolution. Peltier Temperature Controllers (TEC) is used to facilitate the heat dissipation and maintain a constant temperature.

Supplementary Note 4 – Chip Characterisation and Loss Analysis

The calibration is done by applying electrical power to the phase shifter, while measuring the optical power output at the corresponding optical port. The collected data is fitted according to $Y = -a \cdot cos(b \cdot (P + c) + d$, where d is a constant background, a is the maximum amplitude of the signal, b, c are coefficients depicting relationship between phase and supplied power P. The average R^2-values obtained is 0.99 (the best to reach is 1), which indicates that the model adequately reproduces the data observed from the measurements. The average visibility is 99.85%. The extinction ratio of the MZI is about 27 dB. We show the calibration of several exemplary heaters (the 100 μm and 342.8 Ω ones) in Figure 5. Their fitting results are shown in Table. 1. The fitting parameters are reported with a 95% confidence level. By the characterisation, we can tell how much electrical power is required to reconfigure each phase shifter to realize the designed phase.

We then investigate the optical losses of the silicon photonic chip. The main contributors of the optical loss are the coupling loss and the component loss. The losses are calibrated before the experiments. Grating couplers are used to guide light into our chip, and the total coupling loss (at input and output port) is -11.6 dB at 1550 nm. The standard propagation loss of our waveguide (450 × 220 nm^2) is 2 dB/cm. The length of our chip structures is about 8 mm and thus the estimated propagation loss is 1.76 dB. The component loss mainly comes from the MMI which is 0.2 dB each. For a N-mode interferometer, the component loss is 0.2 N for each optical path. Current fabrication technologies support a 100-modes interferometer[21].

Supplementary Note 5 – Input Preparation

The following description details will be based on the case of classifying the nonlinear datasets Circle and Spiral. The decomposition and implementation procedure of input preparation matrix are listed. In all theoretical calculations, the value is accurate to two decimal places.

1. Normalize the input. As input preparation is based on the proportion of different path in the light beam, we first normalize all the combinations of the inputs. For example, the inputs we want to generate is (x_1, x_2), $x_1 \in [-1,1]$ and $x_2 \in [-1,1]$, we first append the bias which is constant *1* into it and normalize it to $\frac{1}{\sqrt{3}}(x_1, x_2, 1)$.
2. Decompose the normalized proportion to phase angles on each phase shifter. The input preparation circuit is as shown in Figure 6a. Suppose the input is $\frac{1}{\sqrt{3}}(-1,1,1)$. The MZIs in use is T_5, T_4 and T_3. The transfer function of each MZI is

$$T_k = ie^{i\theta_k/2}\begin{bmatrix} e^{i\phi_k}sin(\theta_k/2) & e^{i\phi_k}cos(\theta_k/2) \\ cos(\theta_k/2) & -sin(\theta_k/2) \end{bmatrix}, k = 1, \dots, 28$$

   As the input is $\begin{bmatrix}1\\0\end{bmatrix}$, the analytical output we got by T_5 is $\begin{bmatrix} ie^{i\theta_5/2}e^{i\phi_5}sin(\theta_5/2) \\ ie^{i\theta_5/2}cos(\theta_5/2) \end{bmatrix}$. We temporarily introduce a global compensation phase ϕ_c for easier computation and by solving $ie^{i(\frac{\theta_5}{2}+\phi_c)}cos\left(\frac{\theta_5}{2}\right) = \frac{1}{\sqrt{3}}$, we get $\theta_5 = 1.91$ and $\phi_c = -0.96$. Thus we know that the input of T_4 from down port is $ie^{i(\frac{\theta_5}{2}+\phi_c)}cos\left(\frac{\theta_5}{2}\right) = \frac{\sqrt{2}}{\sqrt{3}}$. then by solving $\frac{\sqrt{2}}{\sqrt{3}} \cdot e^{i\phi_5} \cdot ie^{i(\frac{\theta_4}{2})}sin\left(\frac{\theta_4}{2}\right) = \frac{1}{\sqrt{3}}$, we get $\phi_5 = 0.79$ and $\theta_4 = -1.57$. The down port input of T_3 is $\frac{1}{\sqrt{3}}$. By solving $\frac{1}{\sqrt{3}} \cdot e^{i\phi_4} \cdot ie^{i(\frac{\theta_3}{2})}sin\left(\frac{\theta_3}{2}\right) = \frac{1}{\sqrt{3}}$, we get $\phi_4 = 4.71$ and $\theta_3 = -3.14$.

3. Implement the phase angles onto the chip. For a binary input with bias $\frac{1}{\sqrt{3}}(-1,1,1)$, The MZIs in control is T_5 , T_4 and T_3 with $(\theta_5, \phi_5) = (1.91, 0.79), (\theta_4, \phi_4) = (-1.57, 4.71), \theta_3 = -3.14$. The global phase does not affect the chip performance. By configuring the subsequent MZIs to form an identity matrix, we can measure the goodness of the input signals we prepared. For better showing, we scan the input (x_1, x_2), $x_1 \in [-1,1]$ and $x_2 \in [-1,1]$ by a step of 0.1 and show in Figure 6b. In output acquisition, each data point is averaged over 50 samplings.

The theoretical resolution of phase modulation is determined by the resolution of the current source. With a minimal increment of 370 nA, the theoretical resolution of phase modulation is 4×10^{-9} rad based on the calibration curve. Although we expect the implementation of phase shifts to be highly accurate, in actual experiments the accuracy of phase shifts deviates from the best-case scenario according to the physical components and signal distortion is unavoidable. The phase resolution is difficult to be directly measured, and we infer it from the photodetection resolution. The normalized photodetection noise is $\sigma_D \approx 0.6\%$ based on our observation. Therefore, the detection limit ($3\,\sigma_D$) of the photodetection is ~ 0.02. The output intensity of an MZI with respect to its inner phase shifter is sinusoidal. Under the small-angle approximation of $\sin(\theta) = \theta$, the phase resolution $\Delta\theta = 0.02$ rad. Therefore, the smallest and distinguishable (through photodetection) phase shift we can implement is 0.02 rad. The limited resolution of intensity detection and phase detection mean that if an event lies within 0.02 of the boundaries established by the hyperplane separating outputs “1” and “0”, it is possible that misclassification will be leaded.

We load the phase information from the ML dataset stored in the local memory. The modulation speed of the input encoding part is limited by the modulation speed of thermo-optic modulators which is ~10 kHz. In our architecture, an N-dimensional input vector requires N MZIs. The scaling problem with speed and power efficiency do exist with our current proof-of-principle chip design if it scales to hundreds of modes. To improve the power efficiency and operation speed, we consider adopting advanced modulators in future chip design, such as the high-speed (26 Gbit/s) and ultra-low-power (3.8 mW) carrier-depletion modulator[22]. We can also potentially mitigate the power scaling problem with other types of modulators, such as the ones based on phase change materials[23] or ultralow power MEMS phase shifters[10]. Trade-off between the speed and power efficiency will always be present.

From the perspective of the circuitry design, we have a substitute for the universal linear optical circuit to reduce the required number of MZIs, namely the Fast approximation design. Researchers from the machine learning community have affirmed the usefulness of Fast approximation of rotations in network constitution and training[24]. The Fast design reduces the required computational resources by a large extent. The universal design consists of $N(N-1)$ training parameters, whereas the Fast design consists of only $N\log_2(N)$ matrices which is the minimum cost to allow all input coordinates to interact with each other. This design is certainly non-universal, but one can possibly find the approximation of the optimal weight matrix in the subspace formed by the Fast design when training a neural network, which significantly reduces the training space and eases the burden of training process.

Supplementary Note 6 – Decomposition and Implementation of Complex Weight Matrices

The decomposition of weight matrices is similar procedure as input preparation. Here we give an explicit example of decomposing and implementing the weight matrices. We denote the weight matrices for classifying the dataset Spiral as

$$\mathrm{W}_1 = \begin{bmatrix} -0.89 - 0.87\mathrm{i} & -0.39 + 8.37\mathrm{i} \\ 7.01 + 5.60\mathrm{i} & 6.43 + 4.63\mathrm{i} \end{bmatrix}, \mathrm{b}_1 = \begin{bmatrix} 4.76 + 3.92\mathrm{i} \\ 0.78 - 2.65\mathrm{i} \end{bmatrix}$$

$$\mathrm{W}_2 = \begin{bmatrix} 2.83 - 2.71\mathrm{i} & -5.09 + 4.91\mathrm{i} \\ 2.20 - 2.11\mathrm{i} & 6.76 - 6.14\mathrm{i} \end{bmatrix}, \mathrm{b}_2 = \begin{bmatrix} -9.24 + 8.85\mathrm{i} \\ -17.12 - 1.04\mathrm{i} \end{bmatrix}$$

1. Normalization of weight matrices. The weight matrices implemented onto the chip should satisfy that its norm is no larger than *1*. It is easy to understand as the chip is passive and the optical power can only decay and cannot increase. Be careful that the bias of subsequent layers should be scaled according to the normalization factors of the previous layer. By including bias into the weight matrices and normalization, we get

$$W_1 = \begin{bmatrix} -0.06 - 0.06i & 0.52 + 0.41i & 0.17 + 0.14i \\ -0.02 + 0.62i & 0.47 + 0.34i & 0.02 - 0.09i \end{bmatrix}$$

$$W_2 = \begin{bmatrix} 0.24 - 0.23i & 0.19 - 0.18i & -0.02 + 0.02i \\ -0.44 + 0.42i & 0.58 - 0.53i & -0.05 - 0.00i \end{bmatrix}$$

2. Singular vector decomposition. As the transfer matrix of a linear optical circuit is unitary, we need to decompose the arbitrary complex-valued matrix into the product of a unitary matrix, a diagonal matrix and another unitary matrix like $\mathrm{S} = \mathrm{UDV}$. Correspondingly we arrange different areas on the optical neural chip to realize different matrices as shown in Figure 6a.

$$W_1 = U_1 D_1 V_1 \tag{1}$$

$$U_1 = \begin{bmatrix} 0.58 + 0.00i & 0.81 + 0.00i \\ 0.78 - 0.19i & -0.56 + 0.13i \end{bmatrix}$$

$$D_1 = \begin{bmatrix} 1 & 0 & 0 \\ 0 & 0.5010 & 0 \end{bmatrix}$$

$$V_1 = \begin{bmatrix} -0.18-0.44i & 0.09+0.80i & 0.30-0.14i \\ 0.61-0.60i & 0.39-0.15i & -0.26-0.03i \\ 0.14-0.01i & 0.22-0.33i & 0.89+0.05i \end{bmatrix}$$

The same process is executed for W_2.

3. Decomposition into phase angles. The decomposition scheme is as shown in Figure 7 by taking a 6-mode structure as example. The decomposition starts from the rightmost column of MZIs. We suppose that the light is injected from the first input port, then the outputs corresponds to the 6th column in the weight matrix. By this way we can figure out the T_1, T_6, T_{10}, T_{13}, T_{15} and $M_1 = T_1T_6T_{10}T_{13}T_{15}$. By $U= M_1U_1$ and $U_1=M_1^{-1}U$, we can figure out the next column T_2, T_7, T_{11}, T_{14}, so on and so forth to get the phases to reconfigure a designed unitary matrix. Compensation phases are placed before the matrix input and can be implemented to the outer phase shifter of last stage.

4. Chip implementation and validation.

Supplementary Note 7 – On-chip Phase-diversity Homodyne Detection

Phase-diversity homodyne detection determines the cosine and sine of an angle to tell what exactly the angle is within the range of [0, 2π]. The fundamental concept is to take the product of electric fields of the modulated signal light and the reference light[25]. The principle of phase-diversity homodyne detection is shown in Figure 8. One can use commercial 90° optical hybrid circuit. Here, we highlight the realization of the phase-diversity optical homodyning on chip. Since the signal light and the reference light divided from the same source, they have the same frequency and the same polarisation. Suppose the output of the neural network is E_s and the reference light is E_l,

$$E_s(t) = A_s(t)e^{i(w_st+\phi_s)} \quad (2)$$

$$E_l(t) = A_l(t)e^{i(w_l t+\phi_l)} \quad (3)$$

where $A_s(t)$ and $A_l(t)$ are their amplitudes, w_s and w_l are the optical frequencies and $\phi_{s,l}$ the phases. The coherent detection is realized by an MZI. According to the transfer function, the output field is

$$\begin{bmatrix} E_1 \\ E_2 \end{bmatrix} = ie^{i\theta/2}\begin{bmatrix} e^{i\phi}sin(\theta/2) & e^{i\phi}cos(\theta/2) \\ cos(\theta/2) & -sin(\theta/2) \end{bmatrix}\begin{bmatrix} E_s \\ E_l \end{bmatrix} \quad (4)$$

We take the output from the up port and the bottom port respectively,

$$E_1(t) = ie^{i(\phi+\theta/2)}(sin(\theta/2)E_s(t) + cos(\theta/2)E_l(t)) \quad (5)$$

$$E_2(t) = ie^{i\theta/2}(cos(\theta/2)E_s(t) - sin(\theta/2)E_l(t)) \quad (6)$$

The photocurrent is proportional to the square of the input optical signal. When $\theta = \pi/2$, the intensity

$$I_1(t) = \mathcal{R}\{|E_1(t)|^2\} = \frac{1}{2}|A_s(t)|^2 + \frac{1}{2}|A_l(t)|^2 + A_s(t)A_l(t)cos(\phi_s - \phi_l) \quad (7)$$

$$I_2(t) = \mathcal{R}\{|E_2(t)|^2\} = \frac{1}{2}|A_s(t)|^2 + \frac{1}{2}|A_l(t)|^2 - A_s(t)A_l(t)cos(\phi_s - \phi_l) \quad (8)$$

Thus, the cosine component of the relative phase $\Delta\phi = \phi_s - \phi_l$ is obtained. To determine the $\Delta\phi \in [0,2\pi]$, we then add an additional $\pi/2$ phase shift in the reference light to achieve another two outputs,

$$E_3(t) = \frac{\sqrt{2}}{2}ie^{i(\phi+\theta/2)}(E_s(t) + iE_l(t)) \quad (9)$$

$$E_4(t) = \frac{\sqrt{2}}{2}ie^{i\theta/2}(E_s(t) - iE_l(t)) \quad (10)$$

The corresponding photocurrents are

$$I_3(t) = \mathcal{R}\{|E_3(t)|^2\} = \frac{1}{2}|A_s(t)|^2 + \frac{1}{2}|A_l(t)|^2 + A_s(t)A_l(t)sin\Delta\phi \quad (11)$$

$$I_4(t) = \mathcal{R}\{|E_4(t)|^2\} = \frac{1}{2}|A_s(t)|^2 + \frac{1}{2}|A_l(t)|^2 - A_s(t)A_l(t)sin\Delta\phi \quad (12)$$

By applying balanced detector between I_1 and I_2, I_3 and I_4, the common ground can be removed,

$$I_I(t) = I_1(t) - I_2(t) = 2A_s(t)A_l(t)cos\Delta\phi \tag{13}$$

$$I_Q(t) = I_3(t) - I_4(t) = 2A_s(t)A_l(t)sin\Delta\phi \tag{14}$$

By the detection results we can retrieve the relative phase and the magnitude by direct detection of the light intensity. We realize the on chip coherent detection by an MZI, exemplary results of the coherent detection with the signal phase varying are shown. On-chip detections avoid the fluctuating phase caused by fibre components and increase the stability and credibility of the detection.

Supplementary Note 8 – Proposed Multi-layered Optical Neural Network

Our nonlinearity functions are achieved by O/E/O conversion. Concretely, we first convert optical signal to electronic signal, and then apply pointwise activation function and convert the electrical signal back to optical signals. The choice of the activation function is dependent on the detection methods. For instance, intensity-based activations such as $M(z) = \|z\|$ require intensity detection, while other functions such as the hyperbolic tangent and Rectifier linear unit (ReLU) variations require coherent (phase-sensitive) detection. Our proposed multi-layer neural network is assisted by an electrical interface[7, 26-28]. A column of reconfigurable MZIs are used to connect between every two layers. The functionality of the MZIs switches between detection and modulation (see Figure 9d). An inline photodiode is placed at the cross port of each MZI. The MZIs are originally set to a state that the signal lights are transmitted entirely from the cross port for detection. Activation function is applied to the obtained electrical signals. The MZIs are then

reconfigured to modulate the signal lights according to the results after the activation function is applied. An alternative method is to draw a small portion of the signal light from the current layer for detection and modulate the remaining signal light that will enter the next layer.

Two circuit arrangements, the feedforward way and the recurrent way, are proposed for the strategy as shown in Figure 9. In feedforward way, all layers are tiled on the optical neural chip and an additional column of MZIs is appended to the end of each layer. In each appended MZI, one of the output ports is used for inline monitoring, and the other is used to guide the light to next layer. The phase shifters on these MZIs are modulated to realize the result of the activation function processing the output of the previous layer. In recurrent way, the optical neural chip consists of only a single layer, which is reused each time with a different weight matrix to achieve multiple layers. For a neural network having 5 layers with 8 neurons in each, we can use a chip that integrates 5 optical circuits, each of which has 8 input modes. An alternative approach is to use the same optical circuit to emulate a 5-layer network by iteratively storing its output, re-modulating the inputs, reconfiguring the weights, and repeating these procedures 5 times. In terms of multi-layer extensions, our proposed structure is assisted by an electrical interface as distinct to those all-optical proposals. The cascadability of our device can be evaluated in terms of the standard criteria of isomorphism, gain, physical and noise cascadability[27].

Supplementary Note 9 – Logic XOR Problem and Generalized XOR Problem

As mentioned in the main text, we applied the single complex neuron on toy logic tasks, i.e., the AND, OR, XOR and NAND task. The results of the AND and OR are shown in

Figure 10. Their truth tables are shown in Table 2. The convergence of the single complex-neuron on logic gate tasks are shown in Figure 11. In the logic gate task, the inputs are four fixed binary combinations. We also use the same complex-valued neuron for general XOR problem which accepts random input samples. A generalized XOR problem is defined with multiple random 2-dimensional input samples drawn from Gaussian distribution. If the two components of an input x_1 and x_2 are of the same sign, the neuron output y is targeted as 0, otherwise it is 1. In our setting of binary logic gate, the mapping from outputs to quadrants are predefined so that data points fall into the first and third quadrants would belong to logic "0" and those fall into the second and fourth quadrants would belong to logic "1". The data samples and the decision surfaces formed by a trained complex-valued neuron are shown in Figure 12. Decision surfaces of a single real-valued neuron are also displayed for comparison.

Supplementary Note 10 – The Effect of Imperfect Phase Shift Implementation

The overlap between features of subspecies in dataset *Iris* is visualized in Figure 13. We test a 3-layer real-valued neural network for benchmarking. The training curves are shown in Figure 14**,** with comparison to the training curve of a complex layer. The simulated accuracy is 99.3% by a single complex layer and 97.3% by the 3-layer real network. Decision boundaries of the complex layer in Iris classification are shown in Figure 15, under combinations of any two of the total four features.

We investigate the effect of inaccurate phase shift implementation on the performance of the artificial neural network. The normalized weight matrix is decomposed into phase shifts, and different levels (standard deviation of 5%, 10%, 15%) of Gaussian noise are

added onto when implementing the phase shift to observe the neuron performance. In a single neuron setting, the original trained accuracy without added noise is 96.00%. Under 5%, 10% and 15% noise levels, the accuracies are 95.3%, 94.00% and 88.0%, respectively.

In a neural network with multiple layers being implemented optically, the cumulative error of each layer will affect the final result. We take a 3-layer real-valued neural network as an example to study the cumulative error. The predicted accuracy of the original trained neural network is 97.3%. Each trained layer is substituted with inaccurate optical implementation sequentially. The original weight matrix of the 1st layer is decomposed by SVD to $w_1=u_1s_1v_1'$ for the calculation of the phase shifts required by u_1, s_1 and v_1 respectively. 5% level of Gaussian noise is then applied, and w_1 is reconstructed using the updated phase shifts. The output z from the 1st layer now becomes complex-valued rather than real-valued as expected in the trained model. To make the network perform normally, we keep the sign (positive or negative) of the output and acquire the output sgn[z]||z|| for subsequent computation. Thus, coherent detection is required even in real-valued architecture to determine the sign of the output, unless the outputs of all the layers are constrained to positive value during training. The same operation is then performed on the 2nd layer and the 3rd layer. The confusion matrices of the original trained model and the optical implementations under different levels of noise (5%, 10% and 15%) are shown in Figure 16. As observed from the multi-layer simulation, cumulative error would aggravate the deviation of prediction accuracy in that the prediction accuracy decreases as the number of layers of the photonic neural network increases.

Supplementary Note 11 – Accommodating Inputs with High Dimension

The confusion matrices of testing samples under the five scenarios in the main text is shown in Figure 17. In handwriting recognition, the datasets consist of 28 × 28-pixel grayscale images. The dimensionality of the dataset is much higher than our optical neural chip. And it is impractical to create an optical neural network with 784 input nodes to accommodate all the 784 pixels as inputs. In order to feed our complex architecture with sufficient information while limiting the network size, we propose to encode the high dimensional real inputs into low dimensional complex values to reduce the dimensionality. The method we demonstrate in this paper is to compress the original high-dimensional data with a complex-valued encoder (the input layer), and the input layer is implemented electrically. Although our input layer is done electrically, it is theoretically implementable with our chip by dividing the 784 inputs to 4 inputs a group. However, it requires a heavy workload. We also study an alternative method which is based on *k*-space representation[7, 29]. The *k*-space profiles are complex-valued and mostly concentrated around small k_x and k_y in the centre of the profiles, thus have the ability to reduce the number of input parameter.

The *k*-space is an extension of the concept of Fourier space which represents the spatial frequency information in two or three dimensions of an object. To be more specific, an image is processed by the 2D Fourier transform $c(k_x, k_y) = \sum_{m,n} e^{jk_x m + jk_y n} g(m,n)$, where $g(m,n)$ is the gray scale value of the pixel located at location (m,n) within the image. All these coefficients are complex-valued. The *k*-space profiles are mostly concentrated around small k_x and k_y in the center of the profiles as seen in Figure 18. Hence it is possible to retain most of the information using only small-k components.

Therefore, we can restrict the data to N coefficients with the smallest k, fulfilling the goal of decreasing the input size.

One major benefit of the k-space representation is its ability to reduce the number of input parameters, thereby reducing the size of the neural network. Additionally, this method reduces the required optical chip size as well as the computational resources to perform the training process because the dimension of the neural network dimension does not need to accommodate all the 784-pixel values as inputs. The Fourier representations are complex-valued, which match well with our complex-valued neural network architecture. The pre-processing can be further accomplished optically by Fourier optics. We conduct a numerical simulation to demonstrate that the k-space representation works in a complex-valued neural network and compare it with a real-valued architecture. A simple activation function based on intensity detection $M(z) = ||z||$ is adopted instead of using a linear model. We test the models on the dataset MNIST and Fashion-MNIST and report the results in Table. 3 and Table. 4. The k-space coefficients are fed into an optical neural network consisting of L layers with N neurons in each later, after which an output layer reduces the final output to 10 attributes. In comparison, the input dimension of real-valued architecture is twice that of complex-valued architecture. Concretely, the dimension of weight matrix of complex-valued network is $N \times N$, and that of real-valued network is $2N \times N$.

Supplementary Note 12 – Complex CNN and RNN for Realistic Datasets

We simulate the performance of complex-valued architecture of the advanced convolutional neural network (CNN) on CIFAR10 and SVHN task. We build a complex

model that consists of a convolution layer (depth of 20, kernel of 6 × 6 and stride of 1), a max-pooling layer (kernel of 2 × 2), followed by another convolution layer (depth of 50, kernel of 6 × 6 and stride of 1) and two fully-connected layer. The loss function is negative log likelihood (NLL) loss

$$C = -\frac{1}{n}\sum_{x} y^T \ln(a^L) == -\frac{1}{n}\sum_{x}\sum_{k=1}^{K} y_k \ln(a_k^L)$$

where y is the desired output and a^L is the output of the model. The training algorithm we use is the simplest Stochastic Gradient Descent (SGD) with a learning rate of 0.01, a momentum of 0.9, and a batch size of 64. Both complex-valued CNN and real-valued CNN model are tested. The testing accuracies of the complex-valued CNN and the real-valued CNN on the dataset CIFAR10 after 50 training epochs are 71% and 69%, respectively. The same network architecture on the dataset SVHN obtains 91% with complex values and 89% with real values. More comprehensive study on advantages of complex CNN were reported in C. Trabelsi, et.al, “Deep complex networks” [30].

In addition, we test a complex-valued RNN and its modification complex-valued Gated Recurrent Unit (complex GRU) against real-valued RNN on the human motion prediction task[31]. We use the Human 3.6M (H3.6M) dataset[32], which includes actors performing 15 varied activities such as walking, eating and smoking. We evaluate the Euclidean distance between our prediction and the ground-truth in angle space. Increasing time horizons from 80 to 400 ms are investigated. The comparison of performance (via mean angle error) is shown in Table. 5 with the lowest error underlined. The lower the error, the better the performance of the network. The column of complex GRU is reported by M. Wolter, et.al[33]. The dimensions of real RNN, complex RNN and complex GRU are 1024, 512 and 512,

respectively. It is observed that in most of the cases, the complex-valued architectures have better performances than the real-valued ones. And the complex GRU perform the best.

Supplementary Note 13 – Network Capacity

We define the capacity of a network as the number of real-valued parameters it takes up. Conventional digital electronic computer represents a complex number $x + iy$ using real numbers (x, y) and thereby doubles the number of parameters of each layer: $p_{\mathbb{C}} = 2p_{\mathbb{R}}$. In conventional computer, high-capacity means occupying more memory and processing more computation. However, in coherent optical implementation, the chip requirements of a real-valued layer are the same as that of a complex-valued layer with the same layer size. Besides, as a $n \times n$ complex layer is equivalent to a $\sqrt{2}n \times \sqrt{2}n$ real layer with respect to network capacity, $\mathrm{n}(\mathrm{n} - 1)$ variables are taken in a complex chip while $\sqrt{2}\mathrm{n}(\sqrt{2}\mathrm{n} - 1)$ are taken in a real one. The difference between the variables $\Delta p_{\mathbb{R}-\mathbb{C}} = \left(\sqrt{2} - 1\right)n^2 + (1 - \sqrt{2})n$ grows quadratically with the layer size n. The difference between the occupied optical chip size of equivalent real and complex network indicates that the complex-valued architectures are more practicable with the size of hidden layer increasing.

Supplementary Note 14 – Complex-valued Nonlinear Activation Functions

The choice of non-linearity activation function is important in the formulation of a neural network. Several activation functions have been proposed in literature for dealing with complex-valued neural networks[34, 35]. In optical implementations, these functions are related to the measurement methods. Identity (or no activation function) requires no measurement at all and can help us identify the tasks which are not linearly separable in

real domain but separable in complex domain. Magnitude-based activations require intensity detection. Other functions require phase-sensitive detection such as the hyperbolic tangent and Rectifier linear unit (ReLU) variations[30, 36]. The Relu variations include ModReLU, CReLu and ZReLU. Among which, the ModRelu is commonly used and chosen in our experiments.

- Identity

$$I(\mathrm{z}) = z \tag{15}$$

- Magnitude

$$M(z) = \|z\| \tag{16}$$

- Hyperbolic tangent

$$\tanh(z) = \frac{\sinh(z)}{\cosh(z)} = \frac{e^{z}-e^{-z}}{e^{z}+e^{-z}} = \frac{e^{2z}-1}{e^{2z}+1} \tag{17}$$

- ReLU variations

$$\mathrm{ModReLU}(z) = \mathrm{ReLU}(|z|+b)e^{i\theta_z} = \begin{cases} (|z|+b)\frac{z}{|z|} & \text{if } |z|+b > 0, \\ 0 & \text{otherwise,} \end{cases} \tag{18}$$

$$\mathrm{CReLU}(z) = \mathrm{ReLU}(R(z)) + i\mathrm{ReLU}(I(z)) \tag{19}$$

$$\mathrm{ZReLU}(z) = \begin{cases} z & \text{if } \theta_z \in [0, \pi/2], \\ 0 & \text{otherwise.} \end{cases} \tag{20}$$

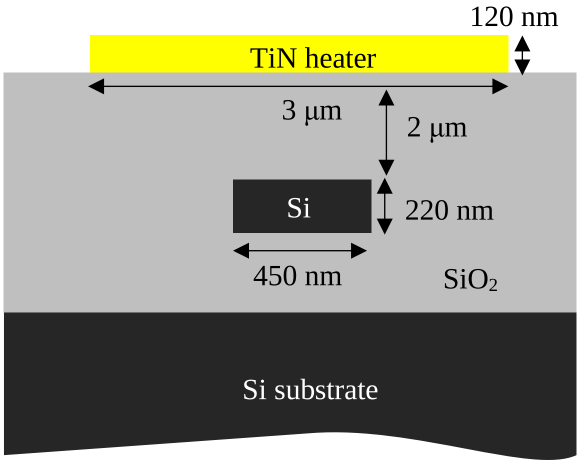

Figure 1 | Cross-section of the waveguide. The waveguide is 450 × 220 nm^2, the TiN heater has length of 100 μm, width of 3 μm and thickness of 120 nm. The distance between TiN heater and the top of waveguide is 2 μm.

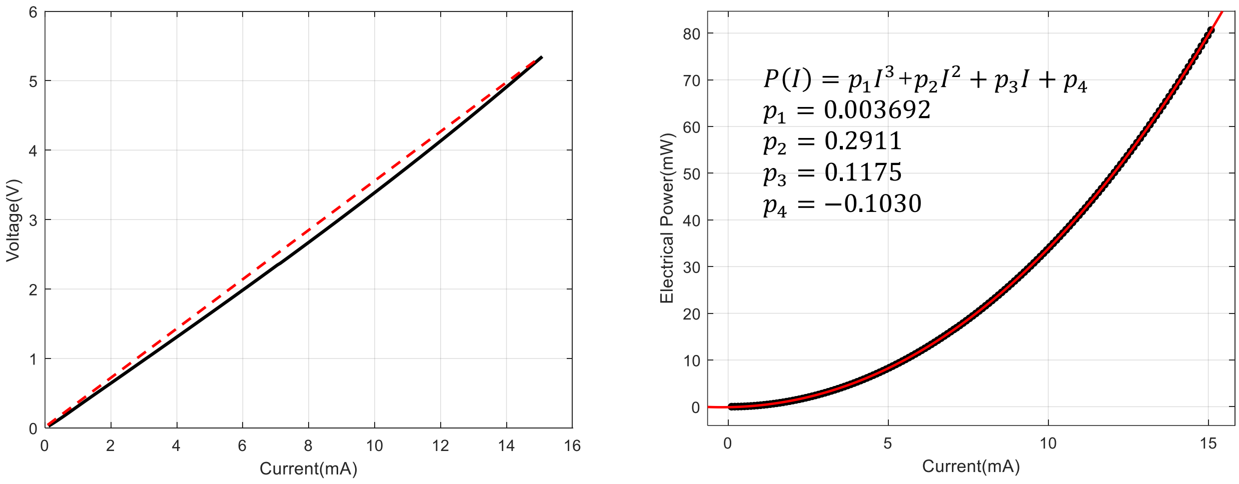

(a) (b)

Figure 2 | Voltammetry characteristics of equivalent resistance of the integrated heater. (a) The chip-integrated heater is not an ideal resistive load as observed from the I-V characteristics. It can be equalized to a resistor with 342.8 Ω. (b) the polynomial fittings of the relationship between electrical power and applied current on the non-resistive heater. As noticed, coefficient of the cubic term p_1 is negligible.

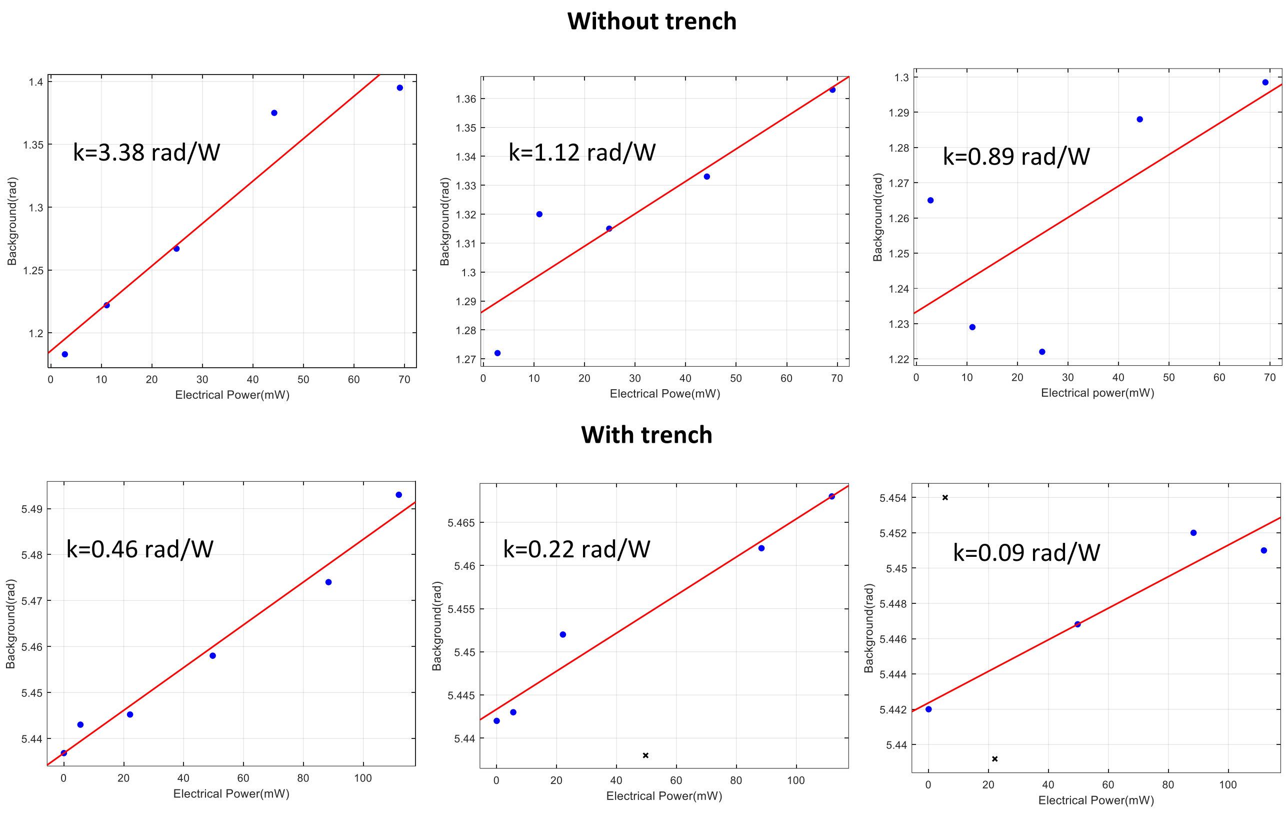

Figure 3 | Isolation Trench helps reducing crosstalk between adjacent heaters. The comparison is made between phase shifters with and without isolation trench. The calibrated crosstalk factor is decreasing with the distance between the neighbour heater (n-heater) and the calibration heater (c-heater) is growing from left to right. For the nearest n-heater, under with trench and without trench, the factor decreases from 3.38 rad/W to 0.46 rad/W.

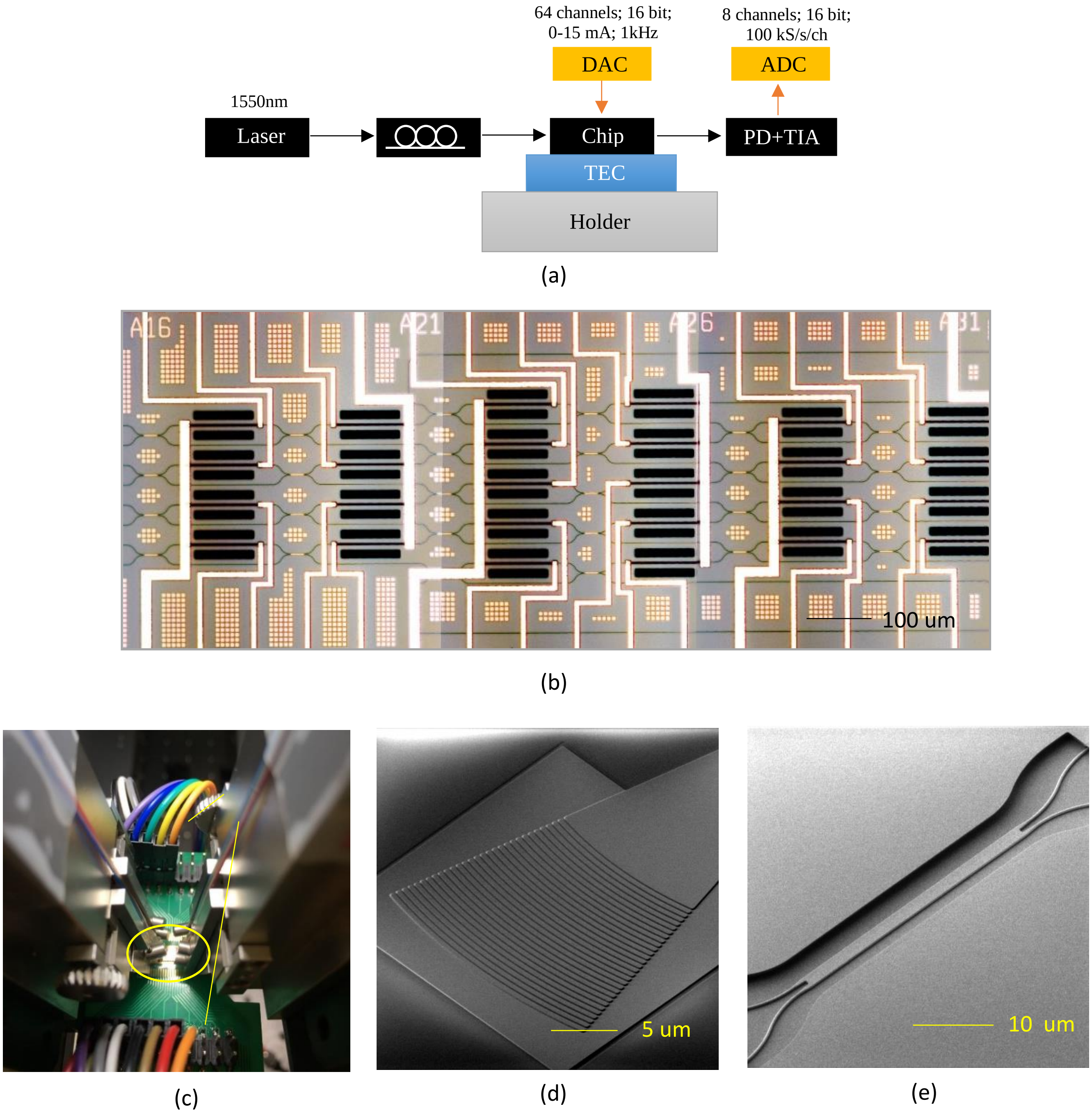

Figure 4 | Electronic control circuits. (a) Main components in the experimental setup. (b) Raw picture of the central part of the chip, displaying the isolation trench. (c) photography of the chip testing bed. (d) and (e) SEM picture of grating coupler and MMI.

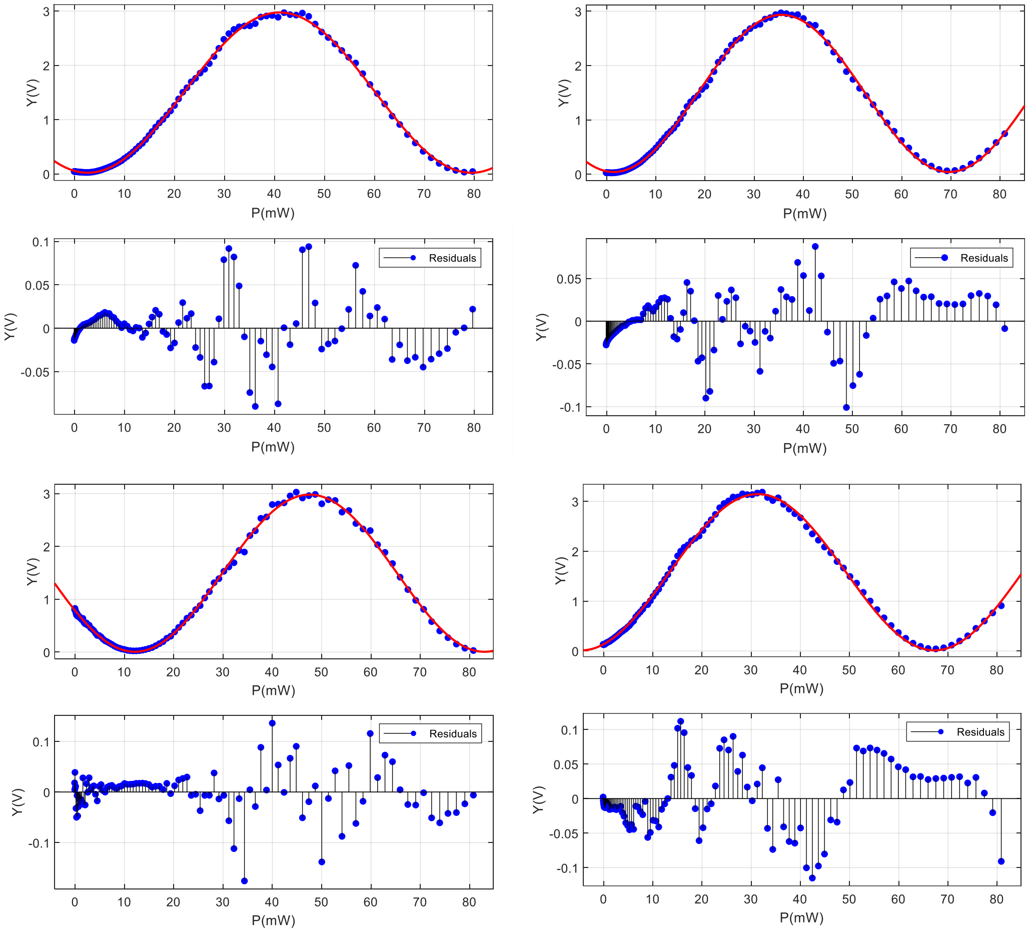

Figure 5 | Fitting curve of the calibration of several exemplary phase shifters. The calibration is done by applying electrical power to the phase shifter, while measuring the optical power output at the corresponding optical port. The collected data is fitted by a sin-like equation. By the characterization, we can calculate out that how much electrical power is required for reconfiguring the phase shifters to realize the designed phases.

Table. 1 | Fitting results of the characterizations of exemplary heaters

	1	2	3	4
a	1.49(1.48, 1.50)	1.45(1.44, 1.46)	1.49(1.48, 1.51)	1.56(1.55, 1.58)
b (×10)	0.82(0.81, 0.82)	0.87(0.87, 0.88)	0.88(0.88, 0.89)	0.87(0.87, 0.88)
c	-0.21(-0.23, -0.19)	0.40(0.38, 0.42)	-1.07(-1.09, -1.05)	0.39 (0.38, 0.41)
d	1.49(1.48, 1.50)	1.45(1.44, 1.46)	1.50(1.48, 1.51)	1.58 (1.56, 1.59)
R^2	0.999	0.998	0.997	0.998
RMSE	0.033	0.044	0.057	0.047
Visibility	99.87%	100%	99.80%	99.18%

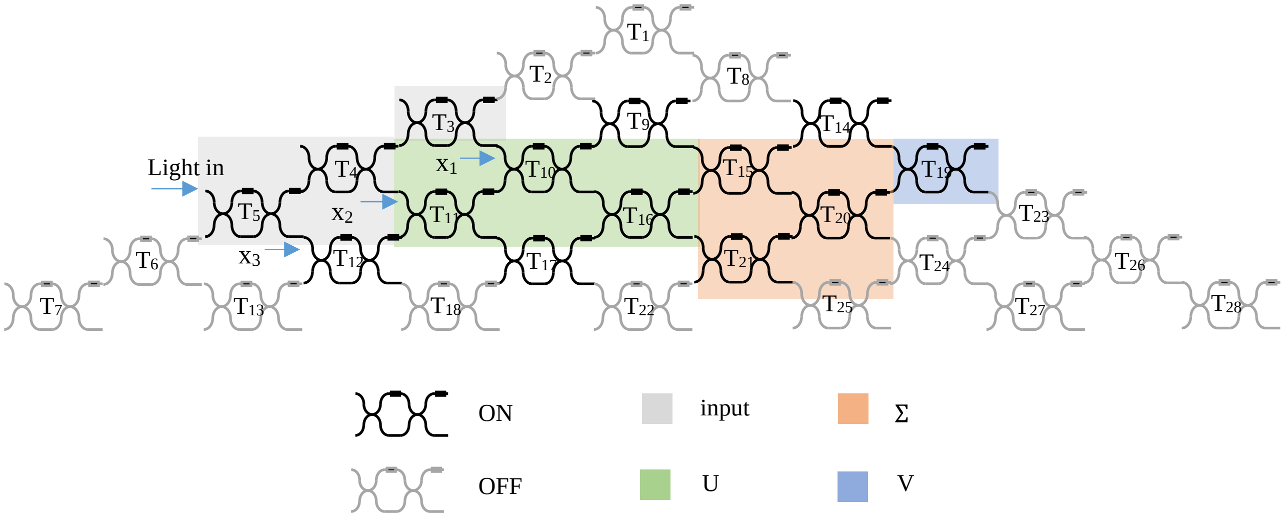

(a)

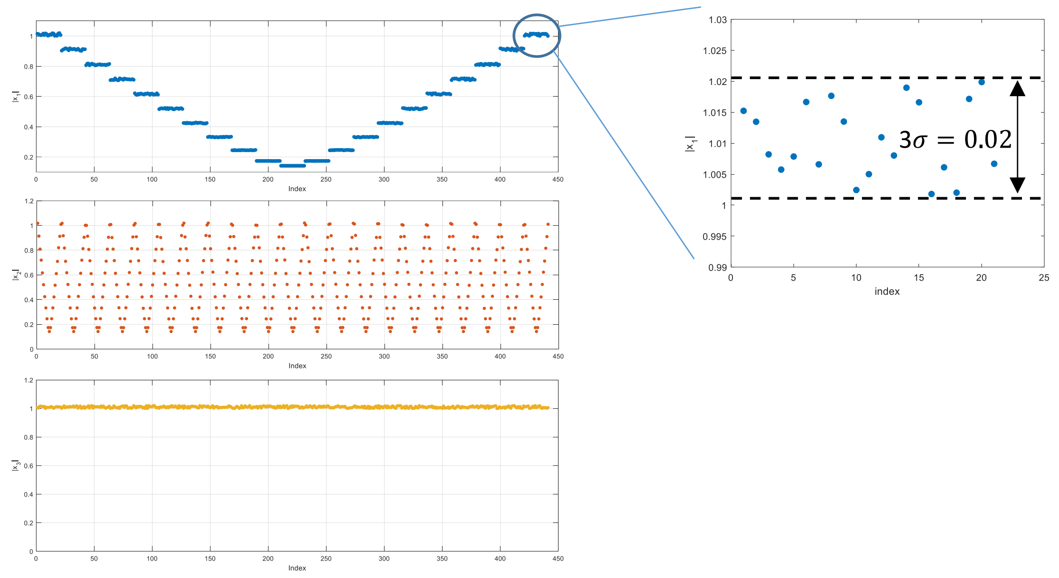

(b)

Figure 6 | Input preparation. (a) The decomposition of chip by several functionality blocks. Each MZI has two working conditions, on and off. MZIs in black are on and those in gray are off. A complex-valued matrix is decomposed by W=UΣV. Three inputs x_1, x_2, x_3 are prepared by MZIs T_3, T_4 and T_5. When measuring the input preparation results, the successor MZIs are configured to be an identity matrix. (b) Measurement results of exemplary binary input vector (x_1, x_2), $x_1 \in [-1,1]$ and $x_2 \in [-1,1]$ by a step of 0.1. x_3 is the constant bias set as 1. Here, we prepare 441(21^2) sets of input signals, which will be used for classification of the nonlinear datasets (Circle and Spiral). The x-axis shows the input index, while the y-axis shows the magnitude of output signal from the three ports. In the zoom in figure of x_1, we obtain the standard deviation σ and the limit of detection $3\sigma = 0.02$.

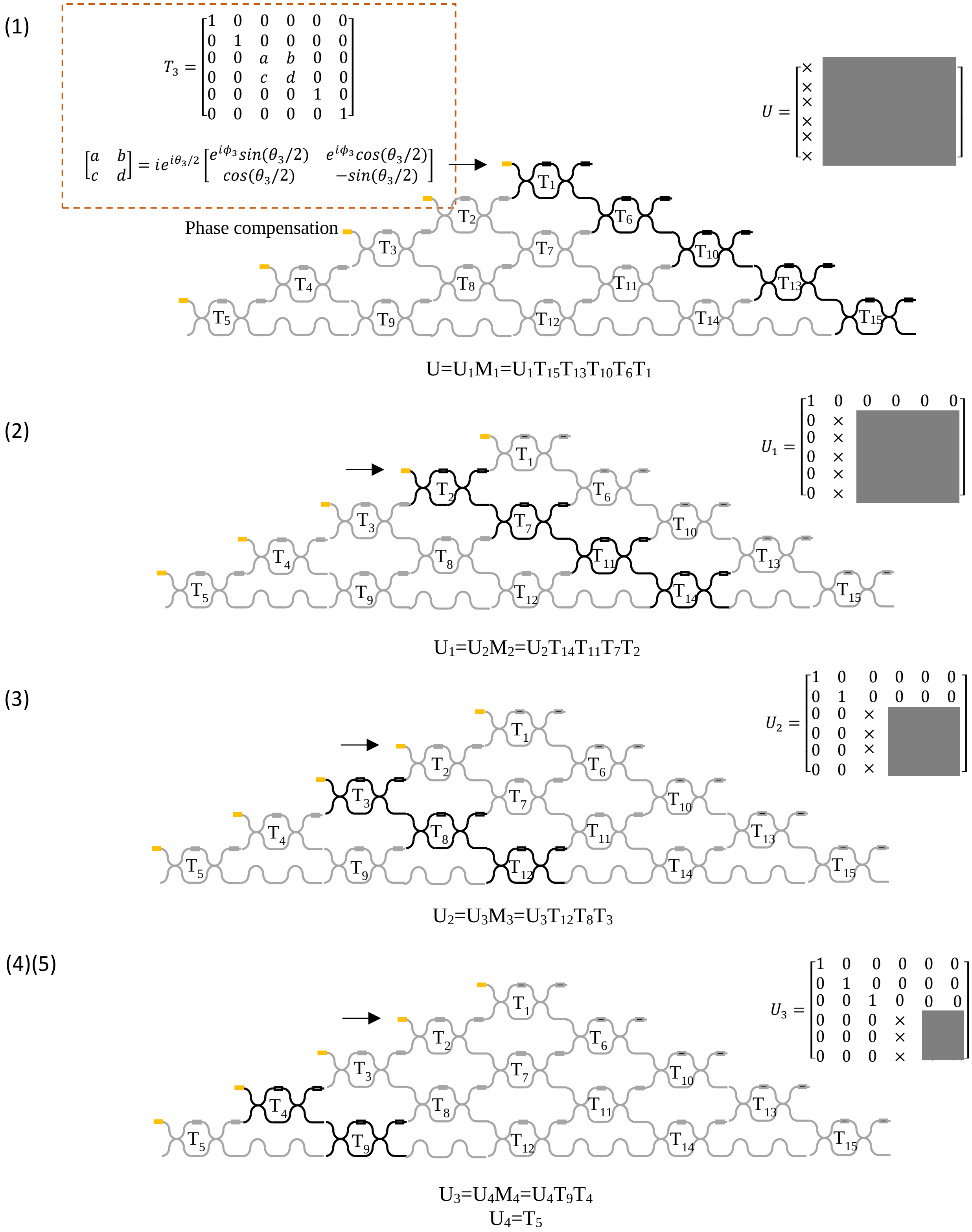

Figure 7 | Decomposition of unitary matrix U. In each subfigure, the black MZIs are being determined. And the " × " in matrices denotes the elements correspondingly being determined. The decomposition starts from the rightmost column of MZIs.

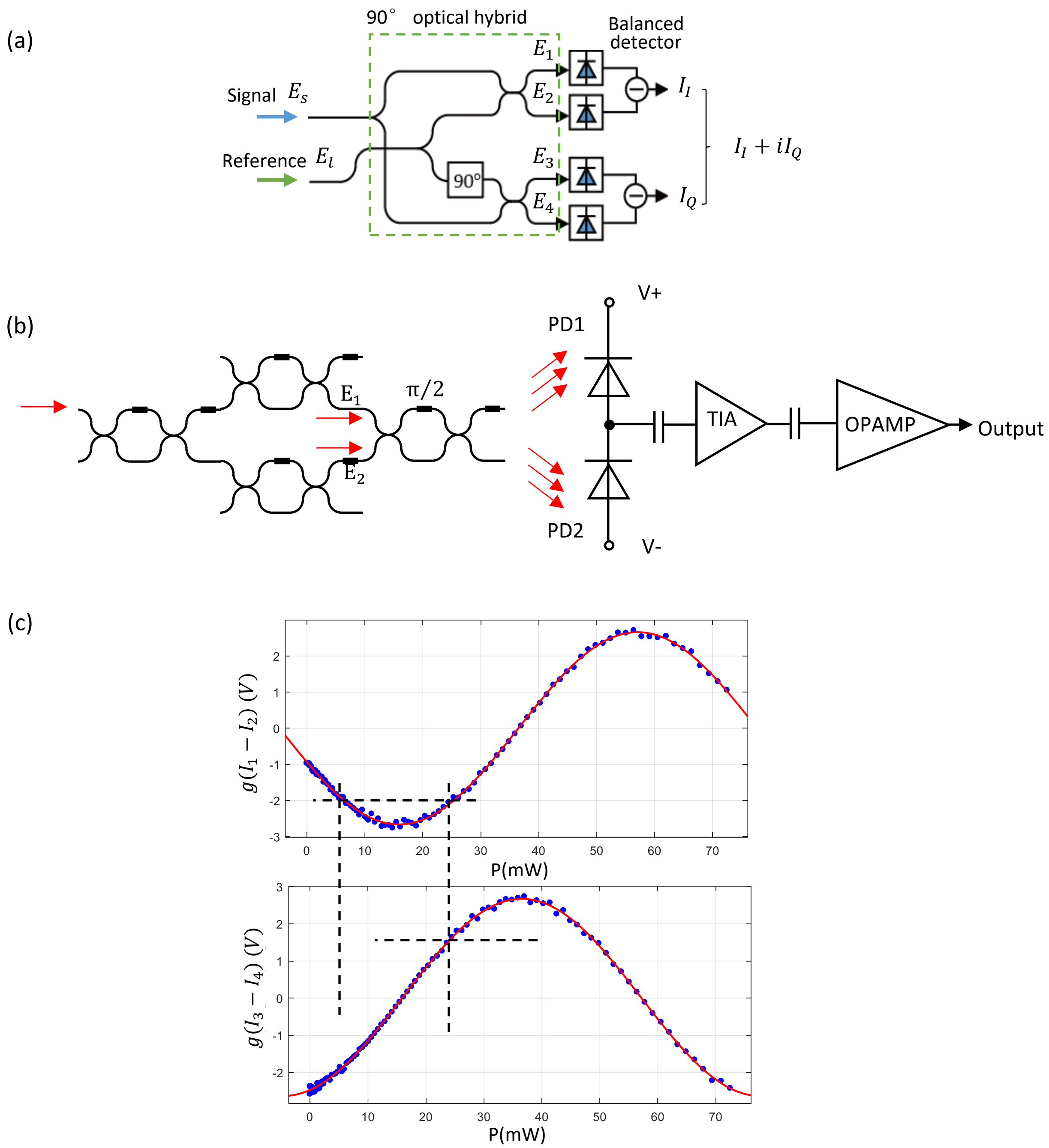

Figure 8 | On-chip phase-diversity optical homodyning. (a) Principle of optical homodyning. (b) On-chip homodyning and balanced detectors. A transimpedance amplifier (TIA) and operational amplifier (OPAMP) is used for the signal amplification. (c) Exemplary results of coherent detection by varying the signal phase, corresponding to equation (13) and (14). For each phase, with its cosine and sine values acquired, the phase can be uniquely determined.

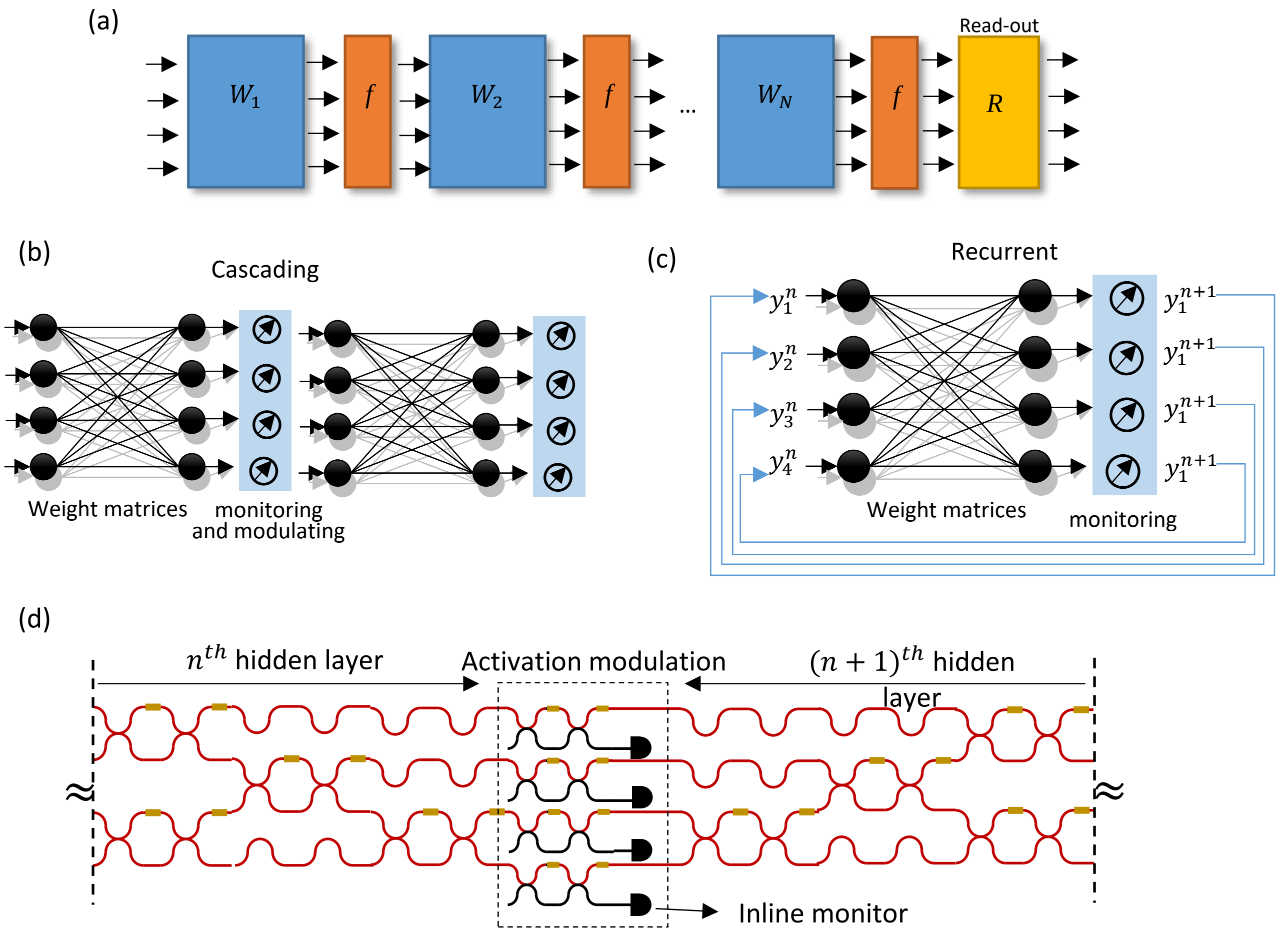

Figure 9 | Multilayered architectures. (a) A general structure of multi-layered neural networks. A nonlinear activation function f is applied to the end of each output signal being transformed by weight matrix W. Two strategies can be used for realizing a multi-layered neural network. (b) Feedforward way, in which all the layers are tiled on the optical neural chip. An additional column of MZIs is appended to the end of each layer for monitoring and modulating. (c) Recurrent way, in which only a single layer is fabricated on chip. The single layer is reconfigured for different weight matrices. The activation function is applied on the electrical signals acquired by detecting the output light. (d) The connection between two optical layers. A column of reconfigurable MZIs is connected between the two optical layers. By reconfiguring the MZI with $\theta = 0$, the signal light is transmitted entirely from the cross port and detected by the inline monitor. Being aware of the output y from the nth hidden layer, we apply the activation function electrically onto y and get y^a. we reconfigure the connection MZI to generate new input y^a for the $(n+1)^{th}$ hidden layer. This is the procedure of a detection-based implementation of activation function.

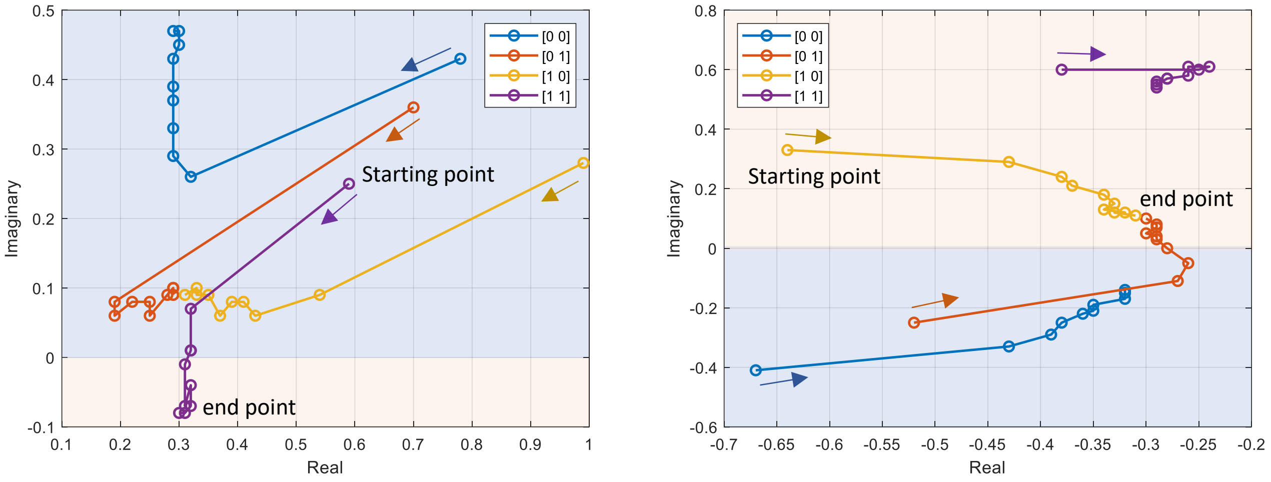

(a) (b)

Figure 10 | Single complex-valued neuron for Boolean task. (a) AND gate. (b) OR gate. 10 iterations are conducted and recorded for each logic gate. Quadrants represent logical “0” are painted blue and those represent logical “1” are painted pink. Being processed by a complex-valued neuron, each of the four different possible combinations of logical inputs converges from a random starting point to the correct end point, via a continuous attenuation of magnitude and phase rotation.

Table. 2 | Truth table of logic gates

A	B	AND (X, Y)	OR (X, Y)	XOR (X, Y)	NAND (X, Y)
0	0	0	0	0	1
0	1	0	1	1	1
1	0	0	1	1	1
1	1	1	1	0	0

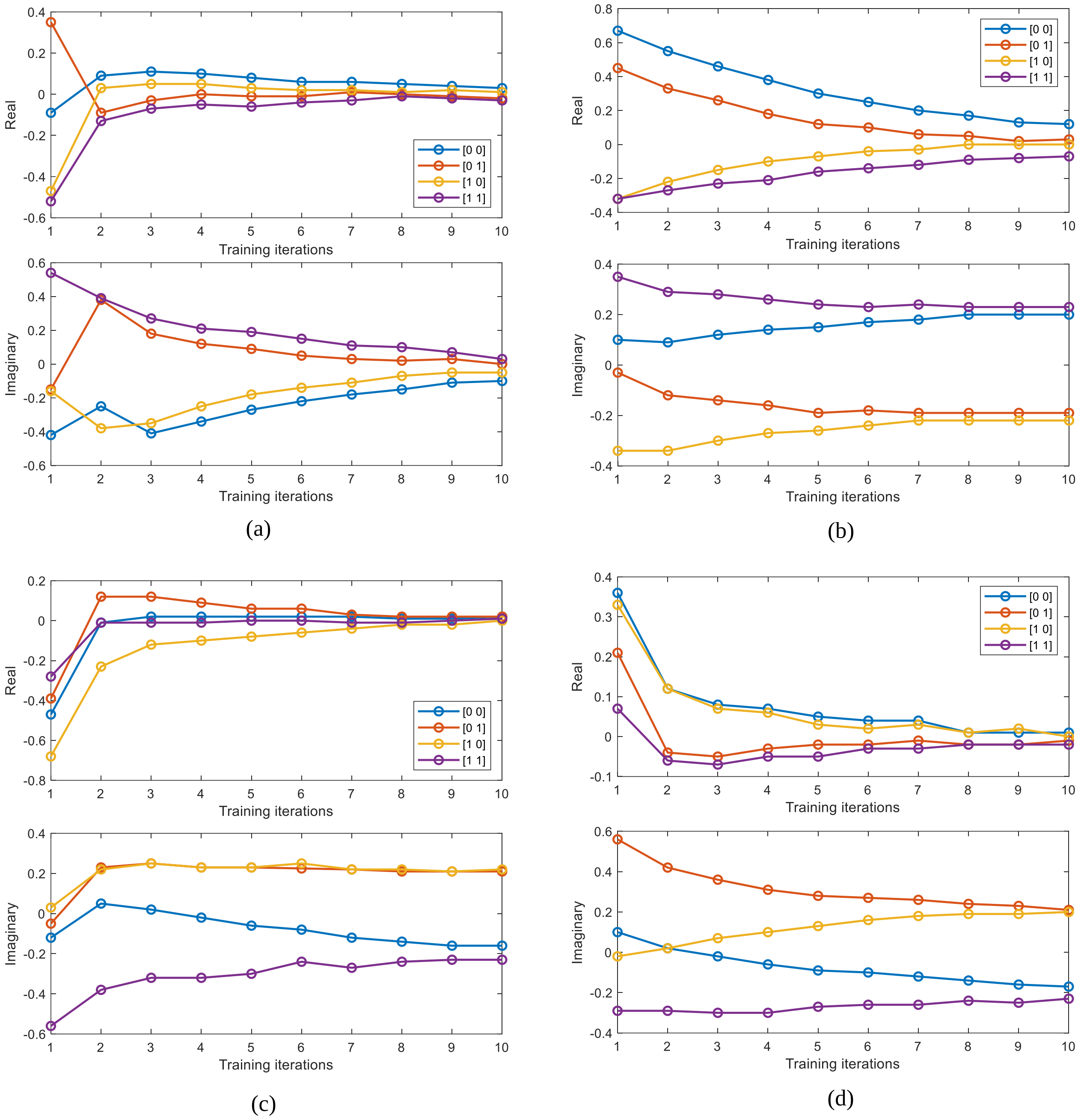

Figure 11 | loss convergence of single neuron for Boolean task. (a) XOR. (b) NAND. (c) AND. (d) OR gate. The loss convergence is shown by the real part and imaginary part of the complex-valued loss. As noticed, XOR is the only symmetry gate (got two "0" and two "1") and the four input combinations converge to separate pre-defined ending points (1+1*j*, 1-1*j*,-1+1*j*,-1-1*j*), thus the loss are converged to zero from both real and imaginary part. For the other three tasks, we observe their convergence, but the converged positions are slightly different from the pre-defined ending points.

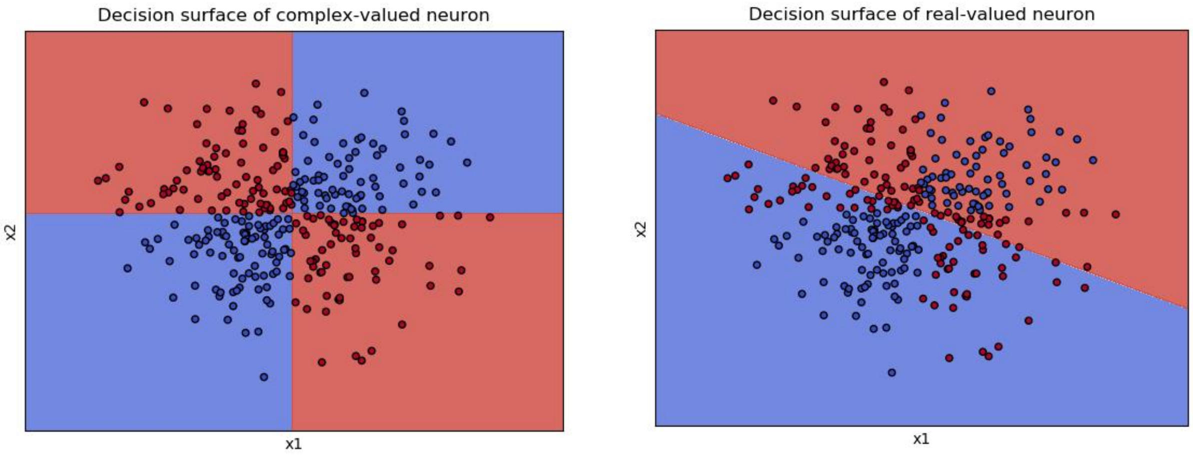

(a) (b)

Figure 12 | Decision regions for general XOR problem. (a) complex-valued neuron and (b) real-valued neuron. A generalized XOR problem is defined with multiple random two-dimensional input samples drawn from Gaussian distribution. If the two components of an input x_1 and x_2 are of the same sign, the neuron output y is targeted as 0, otherwise it is 1. The data samples and the decision regions formed by a trained complex-valued neuron are as shown. Complex neuron is not restricted only to the linear pattern as real neuron is.

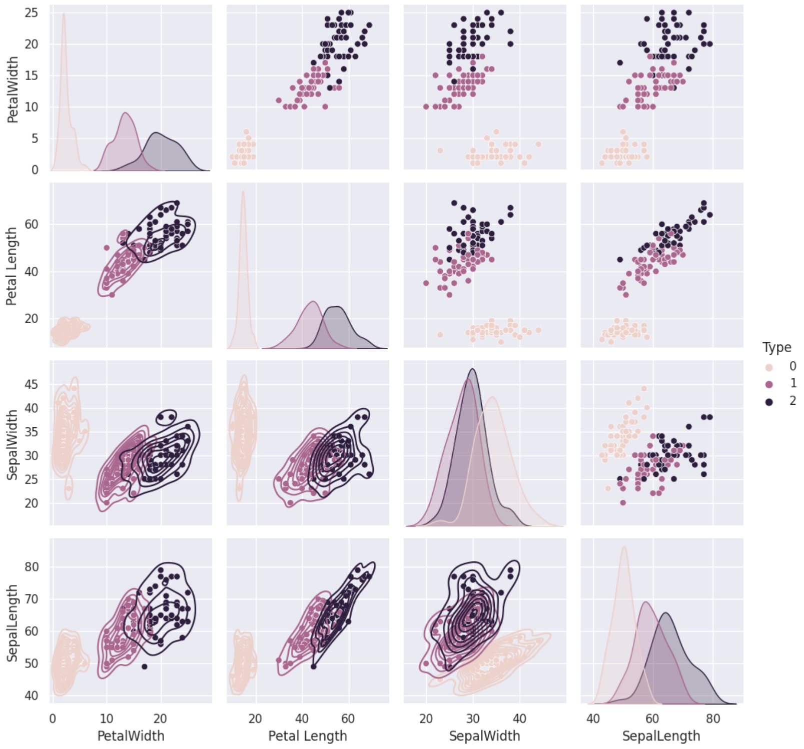

Figure 13 | Dataset visualization by a linear model. The non-triviality of this task is that the three species are indistinguishable by any single one of the four features, with the substantial overlaps between features of the three subspecies shown.

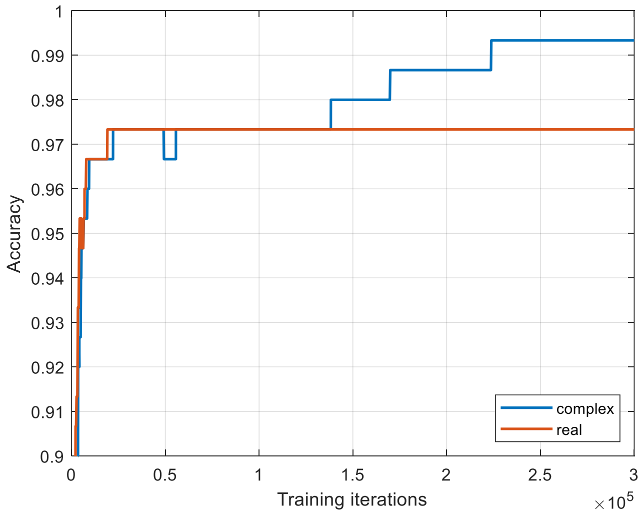

Figure 14 | Training curve of *Iris* classification. Classification accuracy against training iterations are shown for the single complex layer and the 3-layer real network. The complex model achieves a classification accuracy of 99.3%, while the 3 real layers achieves an accuracy of 97.3%.

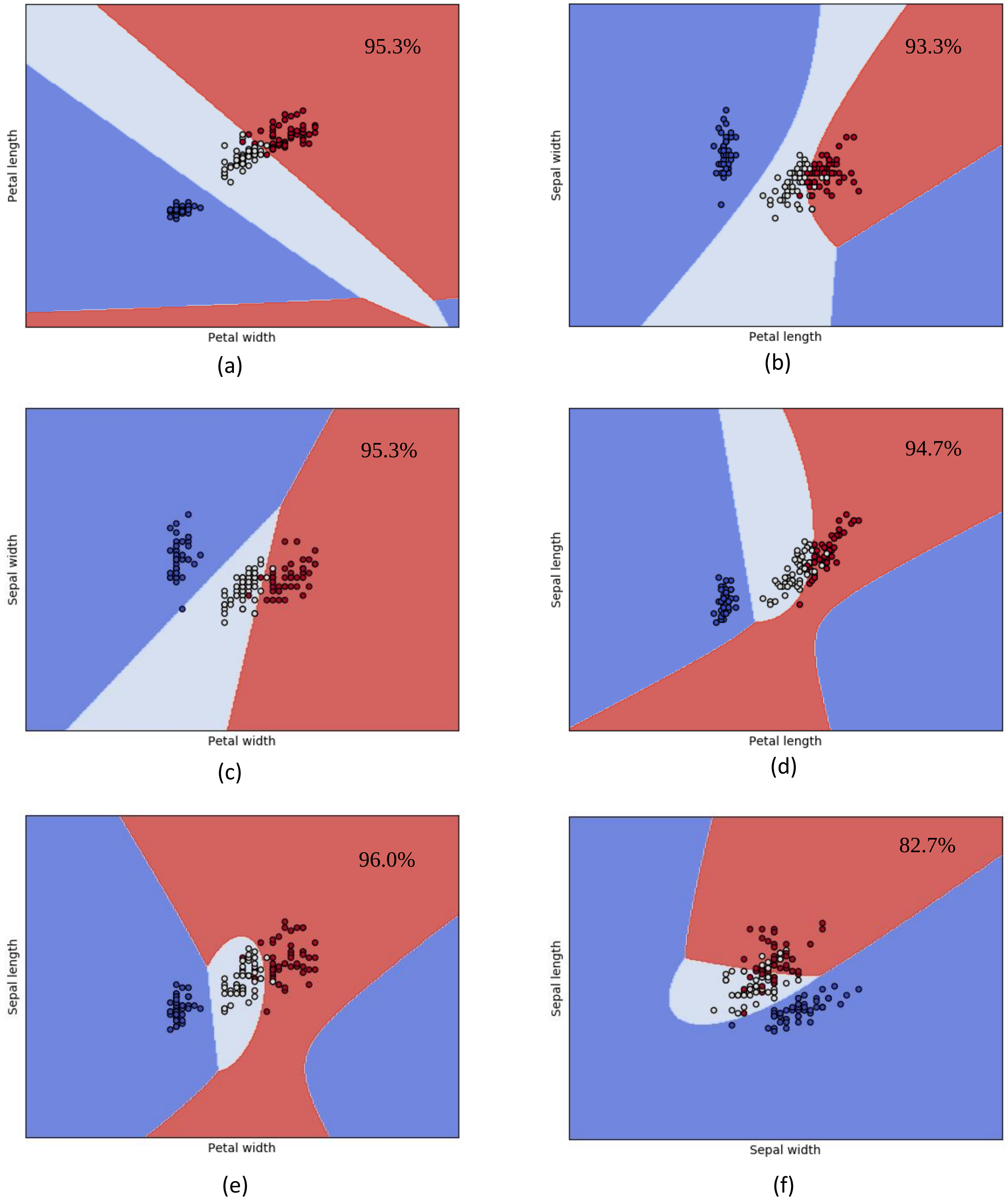

Figure 15 | Visualization of decision boundaries of a complex-valued layer in *Iris* classification. Decision boundaries are shown under combinations of any two of the total four features.

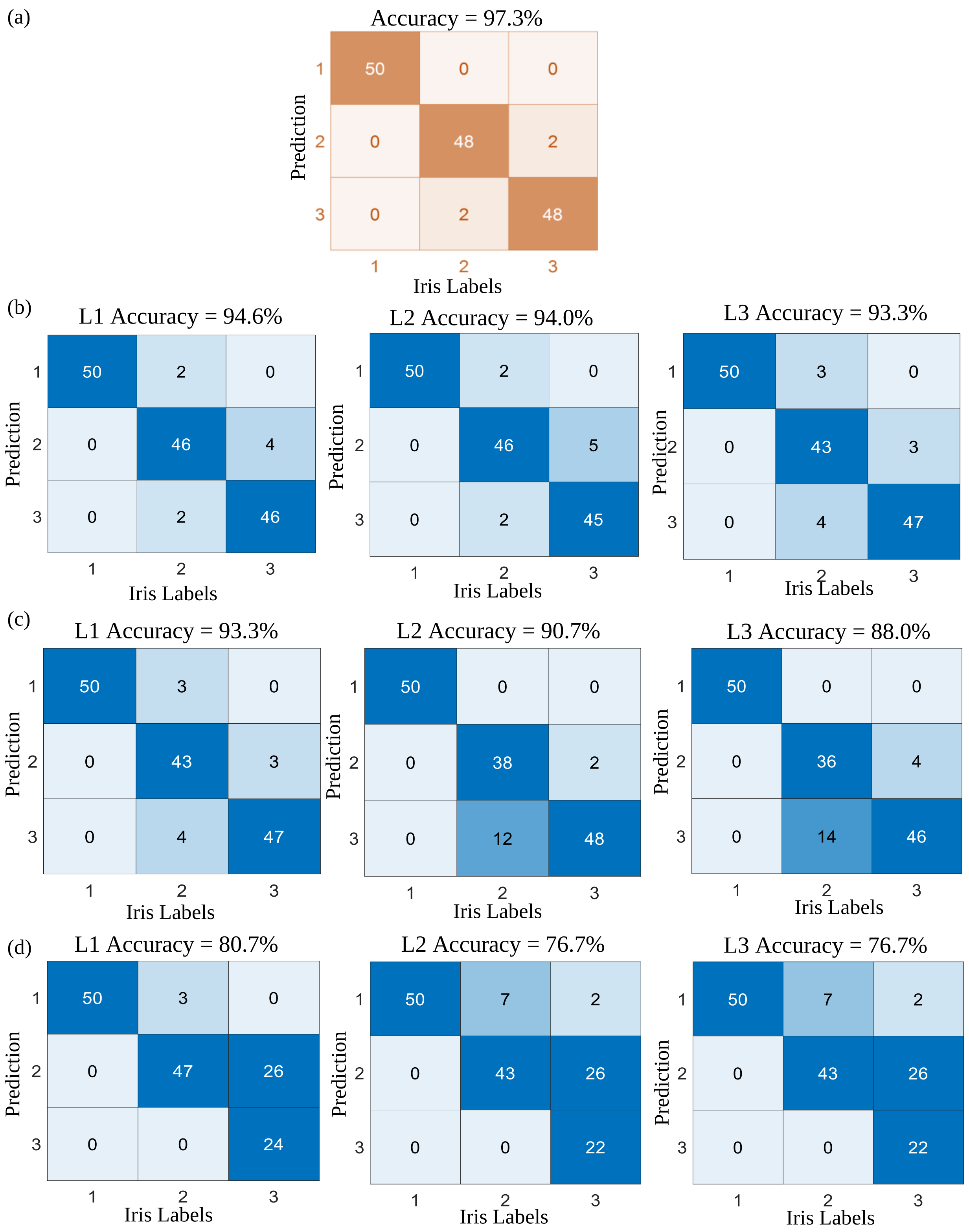

Figure 16 | The impact of the cumulative error with the number of layers increasing. (a) The accuracy of the trained neural network is 97.3%. Noise levels of (b) 5%, (c) 10% and (d) 15% are applied to each layer sequentially.

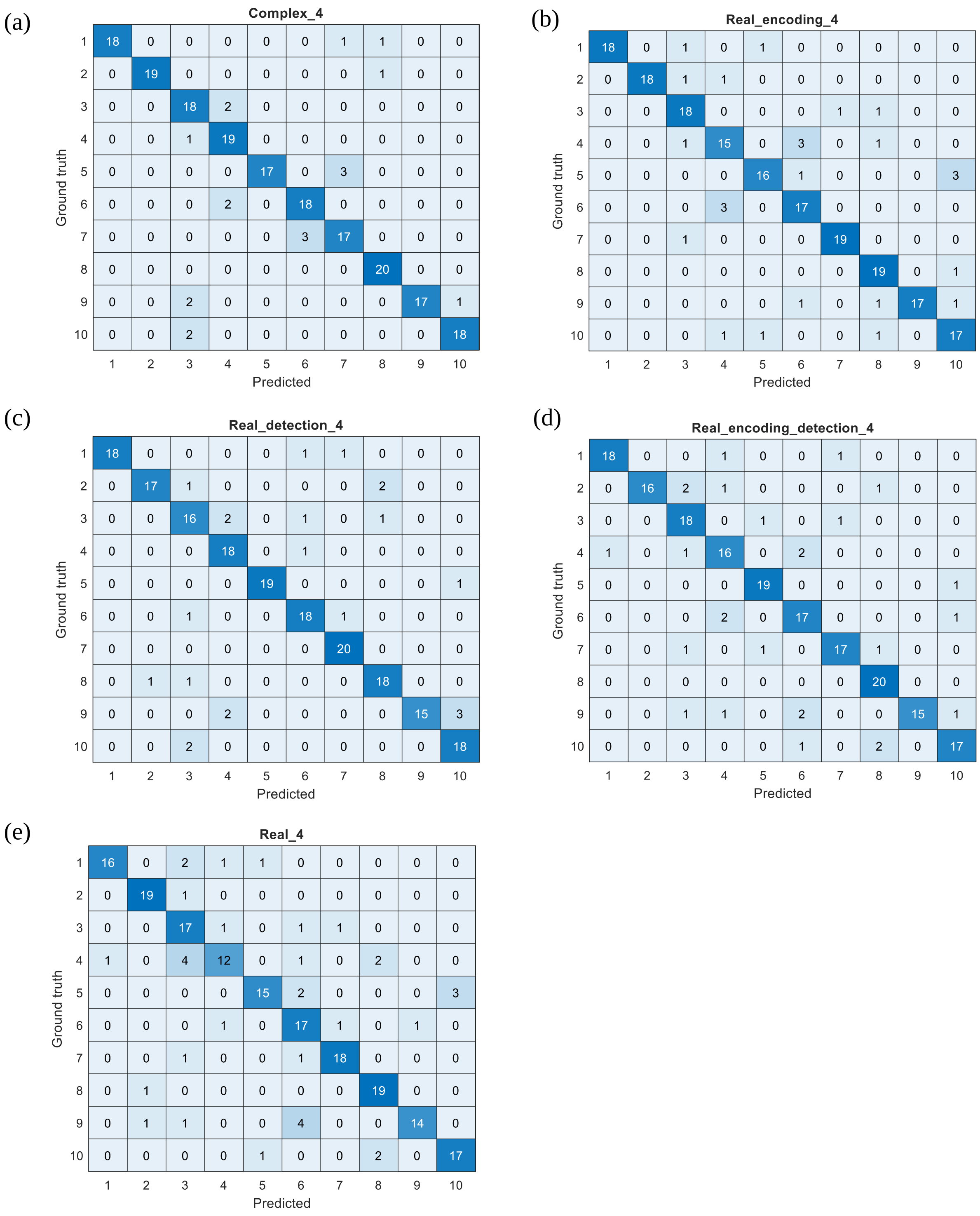

Figure 17 | Confusion matrices under the five scenarios. The accuracies are (a) 90.5% (b) 87.0% (c) 88.5% (d) 86.5% (e) 82.0%.

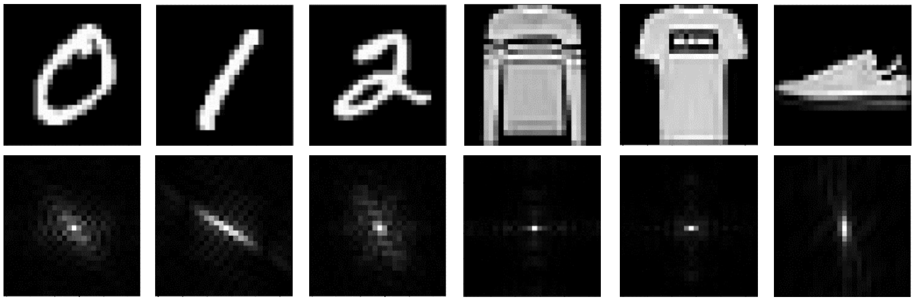

Figure 18 | k-space representation of images from dataset MNIST and Fashion-MNIST. The k-space profiles are complex-valued and mostly concentrated around small k_x and k_y in the centre of the profiles, thus could reduce the number of input parameter.

Table. 3 | Comparison of NLAF in network with L=2 (MNIST)

		Cplx-abs	Cplx-linear	Real-abs	Real-linear
K=N=16	Train_accu	95.66%	90.71%	93.34%	87.52%
	Test_accu	95.06%	89.86%	92.34%	86.98%

Table. 4 | Comparison of NLAF in network with L=2 (Fashion-MNIST)

		Cplx-abs	Cplx-linear	Real-abs	Real-linear
k=N=16	Train_accu	85.24%	80.60%	83.68%	77.89%
	Test_accu	84.19%	79.83%	82.38%	76.94%
k=N=36	Train_accu	88.39%	83.69%	87.57%	80.99%
	Test_accu	86.07%	83.23%	85.70%	80.46%

Table. 5 | Complex RNN, Complex GRU vs. Real RNN on H3.6m dataset (via mean angle error)

	Real RNN (1024)				Complex RNN (512)				Complex GRU (512)			
Action	80ms	160ms	320ms	400ms	80ms	160ms	320ms	400ms	80ms	160ms	320ms	400ms
Walking	0.497	0.620	0.747	0.802	0.370	0.529	0.742	0.809	0.334	0.473	0.690	0.773
Eating	0.389	0.489	0.679	0.835	0.301	0.426	0.649	0.792	0.277	0.411	0.643	0.791
Smoking	0.584	0.776	1.143	1.199	0.433	0.667	1.030	1.171	0.391	0.630	1.039	1.123
Discussion	0.604	0.858	1.077	1.153	0.450	0.733	1.003	1.066	0.456	0.773	1.031	1.092
Directions	0.709	0.898	0.881	0.979	0.520	0.688	0.850	0.958	0.502	0.680	0.843	0.938
Greeting	0.829	1.055	1.370	1.530	0.632	0.902	1.277	1.446	0.610	0.891	1.257	1.410
Phoning	0.755	1.073	1.567	1.695	0.621	1.122	1.523	1.656	0.600	1.128	1.562	1.722
Posing	0.740	0.847	1.346	1.590	0.679	0.945	1.438	1.659	0.612	0.880	1.382	1.619
Purchases	0.773	0.948	1.268	1.351	0.742	0.881	1.168	1.230	0.728	0.894	1.141	1.222
Sitting	0.832	1.037	1.387	1.575	0.579	0.797	1.218	1.429	0.547	0.768	1.185	1.372
Sitting down	1.009	1.301	1.676	1.850	0.609	0.941	1.372	1.564	0.603	0.961	1.409	1.585
Taking photo	0.561	0.775	1.026	1.145	0.342	0.589	0.920	1.069	0.334	0.574	0.893	1.026
Waiting	0.573	0.803	1.214	1.386	0.405	0.656	1.090	1.265	0.389	0.628	1.048	1.223
Walking dog	0.720	0.958	1.223	1.334	0.629	0.913	1.239	1.396	0.652	0.954	1.401	1.484
Walking together	0.475	0.670	0.871	0.919	0.351	0.573	0.826	0.895	0.336	0.562	0.791	0.873
Average	0.670	0.874	1.165	1.290	0.511	0.757	1.090	1.227	0.491	0.747	1.087	1.217

Reference

1. Lin, X. et al. All-optical machine learning using diffractive deep neural networks. *Science* **361**, 1004-1008 (2018).
2. Hamerly, R., Bernstein, L., Sludds, A., Soljačić, M. & Englund, D. Large-scale optical neural networks based on photoelectric multiplication. *Physical Review X* **9**, 021032 (2019).
3. Ishihara, T., Shinya, A., Inoue, K., Nozaki, K. & Notomi, M. An integrated nanophotonic parallel adder. *ACM Journal on Emerging Technologies in Computing Systems (JETC)* **14**, 1-20 (2018).
4. Fiers, M.A.A. et al. Nanophotonic reservoir computing with photonic crystal cavities to generate periodic patterns. *IEEE transactions on neural networks and learning systems* **25**, 344-355 (2013).
5. Freiberger, M., Katumba, A., Bienstman, P. & Dambre, J. Training passive photonic reservoirs with integrated optical readout. *IEEE transactions on neural networks and learning systems* **30**, 1943-1953 (2018).
6. Hornik, K., Stinchcombe, M. & White, H. Multilayer feedforward networks are universal approximators. *Neural networks* **2**, 359-366 (1989).
7. Williamson, I.A. et al. Reprogrammable electro-optic nonlinear activation functions for optical neural networks. *IEEE Journal of Selected Topics in Quantum Electronics* **26**, 1-12 (2019).
8. Shen, Y. et al. Deep learning with coherent nanophotonic circuits. *Nature Photonics* **11**, 441 (2017).
9. Wang, Q. et al. Optically reconfigurable metasurfaces and photonic devices based on phase change materials. *Nature Photonics* **10**, 60 (2016).
10. Jafari, M. & Rais-Zadeh, M. Zero-static-power phase-change optical modulator. *Optics letters* **41**, 1177-1180 (2016).
11. Seok, T.J., Quack, N., Han, S., Muller, R.S. & Wu, M.C. Large-scale broadband digital silicon photonic switches with vertical adiabatic couplers. *Optica* **3**, 64-70 (2016).
12. Edinger, P., Errando-Herranz, C. & Gylfason, K.B. in 2019 IEEE 32nd International Conference on Micro Electro Mechanical Systems (MEMS) 919-921 (IEEE, 2019).
13. Reed, G.T., Mashanovich, G., Gardes, F.Y. & Thomson, D. Silicon optical modulators. *Nature photonics* **4**, 518 (2010).
14. Ziebell, M. et al. 40 Gbit/s low-loss silicon optical modulator based on a pipin diode. *Optics express* **20**, 10591-10596 (2012).
15. Reed, G.T. et al. Recent breakthroughs in carrier depletion based silicon optical modulators. *Nanophotonics* **3**, 229-245 (2014).
16. Harju, A., Siro, T., Canova, F.F., Hakala, S. & Rantalaiho, T. in International Workshop on Applied Parallel Computing 3-26 (Springer, 2012).
17. Karim, M.F. et al. Integration of SiP-Based 60-GHz 4$\,\times\, $4 Antenna Array With CMOS OOK Transmitter and LNA. *IEEE transactions on microwave theory and techniques* **59**, 1869-1878 (2011).
18. He, H. et al. Research on Optical Transmitter and Receiver Module Used for High-Speed Interconnection between CPU and Memory. *Fiber and Integrated Optics* **35**, 212-229 (2016).

19. Absil, P. et al. in 2017 IEEE International Electron Devices Meeting (IEDM) 34.32. 31-34.32. 34 (IEEE, 2017).
20. Shi, T., Su, T.-I., Zhang, N., Hong, C.-y. & Pan, D. in Optical Fiber Communication Conference M3F. 4 (Optical Society of America, 2018).
21. Paesani, S. et al. Generation and sampling of quantum states of light in a silicon chip. *Nature Physics* **15**, 925-929 (2019).
22. Ding, J. et al. Ultra-low-power carrier-depletion Mach-Zehnder silicon optical modulator. *Optics express* **20**, 7081-7087 (2012).
23. Wang, Q. et al. Optically reconfigurable metasurfaces and photonic devices based on phase change materials. *Nature Photonics* **10**, 60-65 (2016).
24. Mathieu, M. & LeCun, Y. Fast approximation of rotations and hessians matrices. *arXiv preprint arXiv:1404.7195* (2014).
25. Kikuchi, K. Fundamentals of coherent optical fiber communications. *Journal of Lightwave Technology* **34**, 157-179 (2015).
26. Ribeiro, A., Ruocco, A., Vanacker, L. & Bogaerts, W. Demonstration of a 4× 4-port universal linear circuit. *Optica* **3**, 1348-1357 (2016).
27. Miller, D.A. Self-configuring universal linear optical component. *Photonics Research* **1**, 1-15 (2013).
28. Miscuglio, M. et al. All-optical nonlinear activation function for photonic neural networks. *Optical Materials Express* **8**, 3851-3863 (2018).
29. Gardner, E. & Derrida, B. Optimal storage properties of neural network models. *Journal of Physics A: Mathematical and general* **21**, 271 (1988).
30. Trabelsi, C. et al. Deep Complex Networks. *arXiv preprint arXiv:1705.09792* (2017).
31. Martinez, J., Black, M.J. & Romero, J. in Proceedings of the IEEE Conference on Computer Vision and Pattern Recognition 2891-2900 (2017).
32. Ionescu, C., Papava, D., Olaru, V. & Sminchisescu, C. Human3. 6m: Large scale datasets and predictive methods for 3d human sensing in natural environments. *IEEE transactions on pattern analysis and machine intelligence* **36**, 1325-1339 (2013).
33. Wolter, M. & Yao, A. in Advances in Neural Information Processing Systems 10536-10546 (2018).
34. Scardapane, S., Van Vaerenbergh, S., Hussain, A. & Uncini, A. Complex-valued neural networks with nonparametric activation functions. *IEEE Transactions on Emerging Topics in Computational Intelligence* (2018).
35. Özdemir, N., İskender, B.B. & Özgür, N.Y. Complex valued neural network with Möbius activation function. *Communications in Nonlinear Science and Numerical Simulation* **16**, 4698-4703 (2011).
36. Arjovsky, M., Shah, A. & Bengio, Y. in International Conference on Machine Learning 1120-1128 (2016).
